# Spatial dynamics of mTOR pathway activity during bovine embryo development

**DOI:** 10.3389/fcell.2026.1766635

**Published:** 2026-02-25

**Authors:** Francieli P. Berling, Viviane B. G. Bacaro, Ricardo I. de Paschoal, Marcella P. Milazzotto, Marcelo D. Goissis

**Affiliations:** 1 Laboratories of In vitro Fertilization, Cloning and Animal Transgenesis, School of Veterinary Medicine and Animal Science, Department of Animal Reproduction, University of Sao Paulo, Sao Paulo, Brazil; 2 Laboratory of Embryonic Metabolism and Epigenetics, Center of Natural and Human Sciences, Federal University of ABC, Santo Andre, Brazil

**Keywords:** differentiation, inner cell mass, nuclear mTOR, phospho-S6, trophectoderm

## Abstract

The mTOR pathway regulates cell proliferation, growth, survival, and metabolism by integrating nutritional and growth factor signaling. In embryos, its activity is influenced by the availability of nutrients in the culture medium, and it can affect the first cellular differentiation event, driving trophectoderm (TE) formation in mice. We hypothesized that mTOR activity is increased in cells poised to become TE and in differentiated TE cells of early bovine embryos. To test this, we assessed mTOR pathway activity through immunofluorescence detection of phospho-S6 (pS6) using confocal microscopy. In morulae, pS6 activity was primarily observed in the outer cells and in early blastocysts, in the TE, while it disappeared in late blastocysts, suggesting a specific pattern for mTOR localization and activity during early embryonic development in bovine.

## 1 Introduction

The union of male and female gametes gives rise to a unicellular embryo, the zygote. At this initial stage, the zygote remains in transcriptional quiescence and depends on maternal factors previously deposited in the oocyte to proceed with development (Vigneault et al., 2009). After successive cleavages, it then undergoes the first cell differentiation event, which determines the inner cell mass (ICM) and the trophectoderm (TE) that will give rise to the fetus and the placenta, respectively (Toyooka, 2020). The biological processes involved encompass several factors and metabolic pathways that modulate cell proliferation, differentiation, and survival.

In this context, in mice, the Hippo pathway acts through the transcriptional cofactor YAP1, which interacts in the nucleus with TEAD4 and TFAP2C to drive the expression of CDX2, the main TE marker (Zhong et al., 2024; Nishioka et al., 2009). Glucose deprivation triggers developmental arrest and prevents CDX2 expression, allowing only expression of transcription factors related to the ICM (Chi et al., 2020). Glucose is required as a metabolic substrate for the pentose phosphate pathway and the hexosamine biosynthetic pathway and also activates the mTOR (mammalian target of rapamycin) pathway, all of which are involved in the differentiation of mouse TE (Chi et al., 2020; Ruane et al., 2020).

The mTOR pathway integrates environmental cues such as nutrient availability, growth factors, and oxygen levels, linking glucose metabolism to Hippo signaling. It acts as a central regulator of cell growth and metabolism (Jewell and Guan, 2013). This signaling occurs through two major multiprotein complexes: mTORC1, which controls biosynthetic processes such as protein and lipid synthesis, and mTORC2, which regulates cell proliferation and survival (Saxton and Sabatini, 2017). Although the prevailing understanding of this pathway is based on these two complexes, recent studies suggest the existence of a third, mTORC3, whose functions and regulatory mechanisms remain unclear (Zhan et al., 2024). Under physiological conditions, mTOR activation promotes controlled cell growth and division; however, its hyperactivation drives uncontrolled proliferation, contributing to the pathogenesis of cancerous cells (Guertin and Sabatini, 2005).

In mice, there is a strong relationship between mTOR activation and TE proliferation (Martin and Sutherland, 2001; Zamfirescu et al., 2021; Cao et al., 2015), as pathway activation induces TFAP2C translation, which interacts with YAP1 and TEAD4 to promote CDX2 expression (Chi et al., 2020). In deer, reduced mTOR activity serves as a central regulator of the paused pluripotent state in mammalian embryos, showing that mTOR inhibition can induce and maintain diapause by suppressing cell growth, anabolic metabolism, and proliferation in the blastocyst (Van Der Weijden et al., 2021). Although glucose removal does not impair TE differentiation and blastocyst formation in bovine embryos (Berling et al., 2024), nutrient reduction in bovine embryo culture media affects TE or ICM cell numbers and mTOR signaling when nutrients are drastically reduced by more than 75% and 50%, respectively (Herrick et al., 2020), suggesting a role for mTOR in this differentiation process. However, there is a lack of knowledge on the spatial dynamics of mTOR activity during early embryo development. Thus, we hypothesized that mTOR activity is increased in cells destined to become TE and in differentiated TE cells of early bovine embryos.

## 2 Materials and methods

### 2.1 *In vitro* embryo production

Oocytes were collected by aspirating follicles measuring 2–8 mm in diameter from ovaries obtained at a commercial slaughterhouse. Grade I cumulus oocyte complexes (COCs) presenting homogeneous cytoplasm and at least threee layers of cumulus cells were selected and matured for 22–24 h in supplemented TCM199 medium TCM 199 (Gibco, Thermo Fisher) supplemented with 10% FBS (Gibco), 22 μg/mL sodium pyruvate, 50 μg/mL of gentamycin, 0.5 μg/mL FSH (Folltropin-V, Vetrepharm), 50 μg/mL HCG (Vetecor, Callier) and 1 μg/mL of estradiol.in 90 µL drops covered with mineral oil at 38.5 °C and 5% CO_2_. After maturation, the COCs were washed and transferred into Fert-TALP medium supplemented with 100 μg/mL heparin, 2 µM penicillamine, 1 µM hypotaurine, and 0.25 µM epinephrine. Frozen–thawed semen was prepared using a Percoll gradient (45/90%) adjusted to a final concentration of 1 × 10^6^ spermatozoa/mL, added to the COCs, then incubated under the same conditions described above. Eighteen hours post insemination (18 hpf), presumptive zygotes were washed, denuded by vortexing, and washed again before being transferred to KSOM (Nagy et al., 2003) medium. Zygotes were cultured in 90 µL drops of supplemented KSOM free of fetal bovine serum (containing 4 mg/mL BSA, essential and nonessential amino acids, and gentamicin) under mineral oil at 38.5 °C in an atmosphere of 5% CO_2_, 5% O_2_, and high humidity. At 90 hpf, feeding was performed by replacing 30% of the culture medium. Compact morulae were collected at 144 hpf, and blastocysts at different stages were collected at 192 hpf. The embryos had their zonae pellucida removed using pronase and were fixed in 4% formaldehyde, washed in phosphate-buffered saline (PBS) containing 1 mg/mL polyvinylpyrrolidone (PVP), and stored at 4 °C until processing.

### 2.2 Immunofluorescence

Fixed embryos were permeabilized with 0.5% Triton X-100 solution in PBS for 30 min and placed in a blocking solution containing 0.1% Triton X-100, 1% BSA, and 10% donkey serum in PBS for 1 h. They were then incubated with primary antibodies anti-GATA3 (AF2605, 5 μg/mL, R&D Systems, United States), anti-phospho-S6 (1,875 mg/mL, D68F8, Cell Signaling Technology, United States), or mTOR (PA1-518, 25 μg/mL ThermoFisher, United States), followed by washes and incubation with secondary antibodies conjugated donkey anti-rabbit AlexaFluor 488 (A21206, 10 μg/mL, Thermo Fisher) or donkey anti-goat AlexaFluor 555 (A21432, µg/mL, Thermo Fisher) for 1 h. Embryos were washed and then counterstained with 100 μg/mL Hoechst 33342 and analyzed using a Leica SPE laser scanning confocal microscope equipped with spectral detection ranging from 430 nm to 720 nm (CAIMI-IB, USP). Total cell number of blastocysts and pS6 fluorescence intensity was determined using ImageJ (Schneider et al., 2012). Fluorescence intensity in embryos was measured in the maximum projection of confocal microscopy images and corrected by subtracting the background fluorescence intensity.

### 2.3 Statistical analysis

Linear regression using PROC GLM of SAS 9.4 (Sas Institute, United States) was performed to compare the total cell number between blastocysts that were positive or negative for anti-phospho-S6 (pS6) staining. Total cell number was considered the dependent variable and presence of pS6-staining was considered the independent variable. To compare pS6 fluorescence intensity among developmental stages, we used the total cell count median to separate blastocysts as early and late blastocysts. ANOVA was performed using PROC GLM of SAS9.4, followed by Tukey’s comparison of means. Fluorescence intensity was considered the dependent variable and embryonic stage was considered the independent variable. Level of significance was set as 5%.

## 3 Results

**FIGURE 1:**
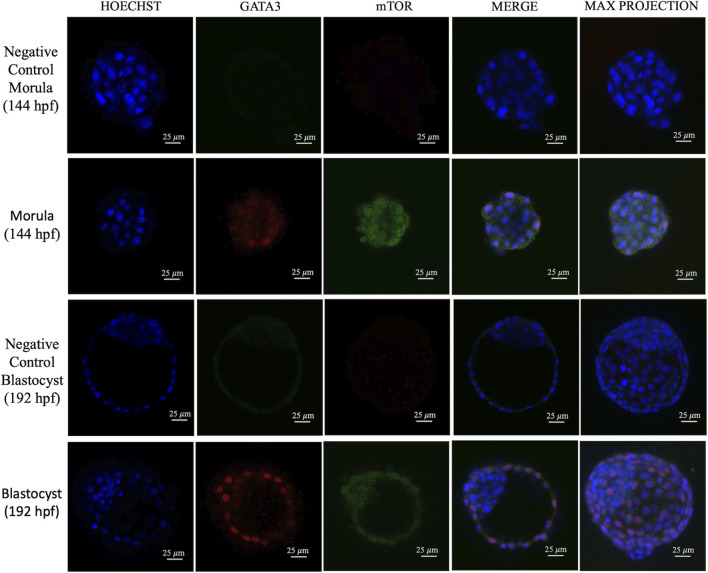
Representative images of embryos immunostained for mTOR and GATA3. Images from confocal microscopy display one representative slice and the correspondent maximum projection. Scale bar = 25 µm. N = 9 embryos.

To analyze the dynamics of the mTOR pathway during early cell differentiation, immunofluorescence labeling of the mTOR protein and its active downstream target, phospho-S6 (pS6) (Meyuhas, 2015), was performed in bovine morulae and blastocysts, combined with the TE marker GATA3 (Home et al., 2009). The mTOR protein exhibited both nuclear and cytoplasmic localization at the morula stage. However, as development advanced to the blastocyst stage, the protein became more restricted to the nucleus (Figure 1).

**FIGURE 2:**
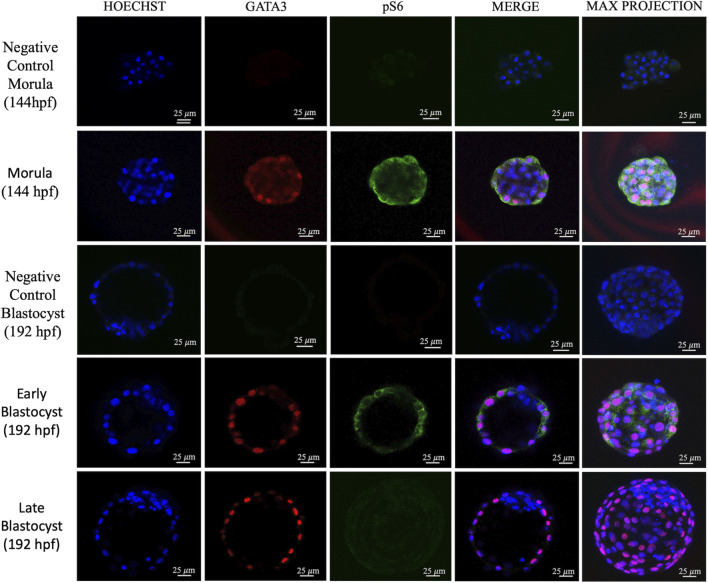
Representative images of embryos immunostained for pS6 and GATA3. Images from confocal microscopy display one representative slice and the correspondent maximum projection. Scale bar = 25 µm. N = 31 embryos.

**FIGURE 3:**
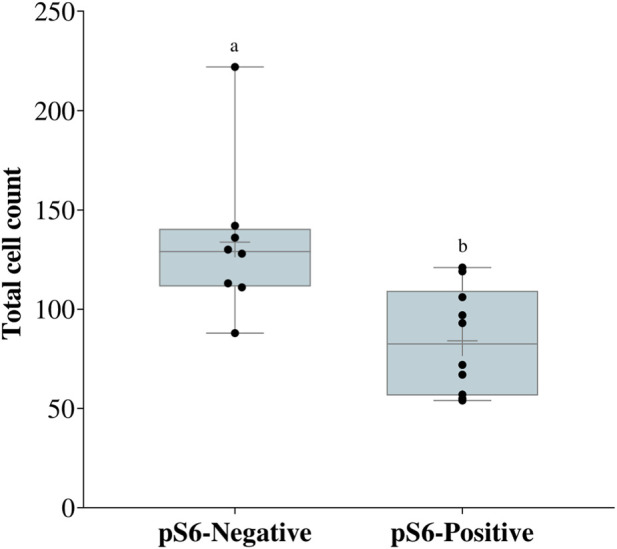
Total cell count of blastocysts after pS6 immunofluorescence. Box-plot of the total cell count from pS6-positive and pS6-negative embryos. Data presented as mean ± SEM (standard error of the mean). Different letters significant statistical difference **(a)** vs **(b)** *p* = 0.02. N = 18 embryos (10 positive, 8 negative).

**FIGURE 4:**
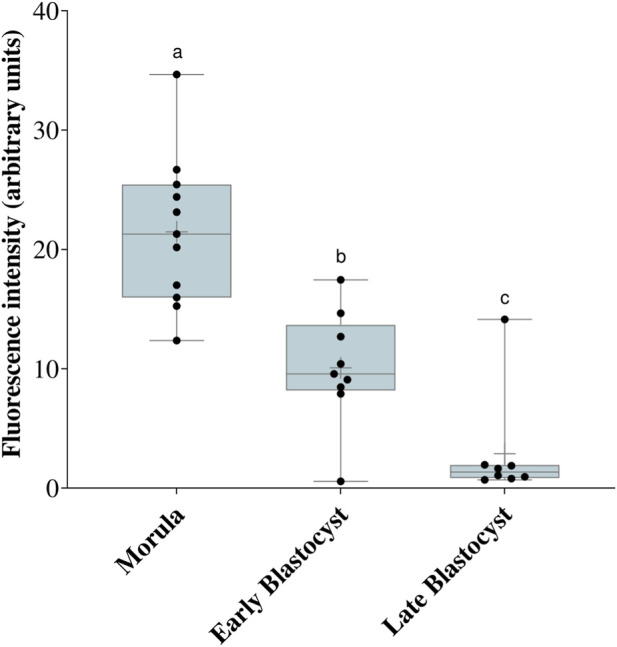
Fluorescence intensity of pS6 in different developmental stages. Box-plot of pS6 fluorescence intensity in morulae, early blastocysts and late blastocysts. Different letters indicate significant statistical difference **(a)** vs. **(b)** p = 0.002, **(a)** vs. **(c)** p < 0.001, **(b)** vs. **(c)** p = 0.02 N = 30 embryos (12 morulae, 9 early blastocyst, 9 late blastocyst).

Considering the activity of the mTOR pathway, increased pS6 staining was observed in the outer cells of morulae, which co-stained with the TE marker, GATA3 (Figure 2). Notably, pS6 staining was present in the TE of some blastocysts, but absent in others. We noticed that apparently larger embryos were negative for pS6 staining (Figure 2). Thus, we counted the total cell numbers of negative and positive embryos and found that indeed the pS6-negative embryos were larger than pS6-positive embryos (Figure 3). We then assessed pS6 fluorescence intensity in different embryo stages: morula, blastocyst (up to the 50th percentile - 106 cells) and late blastocyst (above the 50th percentile). We observed that pS6 intensity decreased as development progressed (Figure 4).

## 4 Discussion

The mTOR pathway is a central integrator of nutritional signals and growth factors that regulate cell growth and metabolism (Saxton and Sabatini, 2017). Studies in mice indicate that its activation is strongly related to the proliferation and differentiation of TE cells (Zamfirescu et al., 2021). This finding converges with the results obtained in knockout mouse embryos for the *mTOR* gene, which showed reduced cell proliferation in the TE and the ICM, negatively impacting gastrulation (Murakami et al., 2004). Also in mice, the sphingolipid pathway also regulates mTOR to control the translation of *TFAP2C* and *CDX2*, thereby inhibiting TE differentiation (Chi et al., 2020). Our study aimed to investigate the spatial dynamics of mTOR activity in bovine embryos, seeking to further understand the role of this pathway in early cell differentiation.

Initially, our study showed both nuclear and cytoplasmic localization of the mTOR protein in morulae, which became more prominent in the nucleus with advancing development to the blastocyst stage, suggesting a specific pattern for mTOR localization and activity in early bovine development. Although predominantly detected in the cytoplasm, mTOR has a nuclear fraction that directly regulates gene expression (Torres and Holz, 2021; Giguère, 2018). This spatial and temporal dynamics could reflect the specific metabolic requirements of each phase. At early stages, cytoplasmic mTOR may act as an immediate nutrient sensor, directing protein synthesis and sustaining the rapid cell proliferation necessary for cleavage and blastocyst formation, whereas its activity should be inhibited to maintain pluripotency in inner cells (Kim et al., 2023). In more advanced stages, the translocation of mTOR to the nucleus signals a transition to genetic-metabolic control, acting as a transcriptional regulator of key gene expression. Nuclear mTOR demonstrates a crucial role as a transcriptional regulator that influences all three classes of RNA polymerases (Pol I, Pol II, and Pol III); more specifically, the transcriptionally regulated target genes of nuclear mTOR overlap with the canonical targets controlled by cytoplasmic signaling (Giguère, 2018).

The literature demonstrates that mTOR signaling is a central regulator of the transition from pluripotency to cell lineage differentiation (Giguère, 2018). In this investigation, although mTOR was detected in all cells of the morula, the mTOR pathway activity observed through pS6 detection occurred only in the outer cells of morulae and later in the TE of early blastocysts. This is in agreement with the observations in mice that demonstrate preferential activation of the mTOR pathway in peripheral cells of morulae (Kim et al., 2023) and also with the suggested role of the mTOR pathway in the specification of the TE as it enables the translation of TFAP2C (Chi et al., 2020), which plays a critical role in the transcription of CDX2 (Cao et al., 2015). Both CDX2 and GATA3 are essential transcription factors for TE specification and maintenance (Ruane et al., 2020), suggesting that the increased pS6 signaling observed in the outer cells and early TE in this work may support the protein synthesis machinery required for these key developmental regulators. Morula-stage embryos undergo intense metabolic and structural reorganization, with a greater demand for protein and membrane synthesis, as well as an increased need for proliferation, higher energy consumption, the onset of polarization, and compaction (Hu et al., 2024), which is consistent with increased mTOR activity at this stage. It is worth mentioning, that the mTOR pathway signals through other effectors such as eIF4E that also regulates translation, and SREBP, which acts on metabolism reviewed by Saxton and Sabatini (2017). Thus, further research is needed to clarify the roles of the mTOR pathway in bovine TE. The inner cells, in contrast to the TE, presented lower mTOR activity, consistent with findings that show lower levels of mTOR activity in undifferentiated stem cells (Cherepkova et al., 2016; Agrawal et al., 2014).

Interestingly, pS6 is present in the TE of early bovine blastocysts, as in mouse blastocysts (Kim et al., 2023). In both mouse and human embryos, pS6 is present mostly in polar TE cells (Iyer et al., 2024); however, we did not find this particular pattern in bovine embryos. More interestingly, we found that pS6 was lower in larger embryos, which developed quicker than their blastocysts counterparts at 192 hpf. It is possible that the reduced use of glucose by the TE in bovine embryos (Gopichandran and Leese, 2003) is reflected into lower mTOR activity, therefore resulting in an absence of pS6 staining. Another possibility is that after S6 phosporylation, its own activity leads to mTOR shuttling to the nucleus (Torres and Holz, 2021), resulting in the observed nuclear mTOR in late blastocysts.

In conclusion, the spatial dynamics of mTOR localization and activity in bovine embryos suggest that this pathway is active in cells differentiating into TE. The observed changes, including high activity of the mTOR cytoplasmic target pS6 in morulae and early blastocysts and the subsequent translocation of the mTOR protein to the nucleus in later stages, demonstrate a specific pattern of regulation throughout early embryonic development in bovine embryos.

## Data Availability

The datasets presented in this study can be found in online repositories. The names of the repository/repositories and accession number(s) can be found below: https://doi.org/10.17605/OSF.IO/ZES3J.
